# Minimally Invasive or Conventional Sternotomy for Mitral Valve Surgery With Concomitant Surgical Ablation for Atrial Fibrillation: A Comparative Systematic Review

**DOI:** 10.31083/RCM39706

**Published:** 2025-08-21

**Authors:** Robert Kashapov, Alexander Afanasyev, Ravil Sharifulin, Sergey Khrushchev, Pavel Ruzankin, Igor Demin, Alexander Bogachev-Prokophiev

**Affiliations:** ^1^E. Meshalkin National Medical Research Center, Institute of Cardiovascular Pathology Research, 630055 Novosibirsk, Russian Federation; ^2^Sobolev Institute of Mathematics, 630090 Novosibirsk, Russian Federation

**Keywords:** atrial fibrillation, Cox-Maze procedure, mitral valve, minimally invasive surgery

## Abstract

**Background:**

Presently, the availability of single-stage surgical correction of mitral valve disease combined with atrial fibrillation (AF) via a mini-access approach remains limited. Moreover, the comparative effectiveness of this procedure versus conventional sternotomy (CS) remains poorly understood. Thus, this study aimed to conduct a comparative assessment of the efficacy and safety of concomitant mitral valve surgery and AF ablation via a minimally invasive approach (minimally invasive cardiac surgery, MICS group) versus the standard sternotomy approach (CS group).

**Methods:**

An extensive literature search was performed to identify relevant studies. Additionally, for comparative analysis, we included isolated studies where the combined intervention was conducted exclusively via either minimally invasive or CS as the primary access.

**Results:**

Freedom from atrial arrhythmia (AA) for MICS and CS was 94.52% [95% CI 91.52, 96.50] vs. 80.76% [95% CI 67.19, 89.59] and 86.22% [95% CI 80.13, 90.66] vs. 86.33% [95% CI 79.39, 91.19] at 1 and 2 years, respectively, with no statistically significant differences. Meanwhile, cardiopulmonary bypass (CPB) and aortic cross-clamp (ACC) times were significantly longer in the MICS group compared to CS (CPB: 151.50 vs. 120.01 min; ACC: 112.36 vs. 101.43 min; *p* < 0.001). There were no differences in mortality between groups (*p* = 0.709). The rate of pacemaker implantation was significantly higher in the CS group (MICS: 3.32% [95% CI 1.58, 6.87] vs. CS: 5.20% [95% CI 2.80, 9.46]; *p* < 0.001).

**Conclusion:**

This meta-analysis found that the minimally invasive approach was associated with longer CPB and ACC times but a lower rate of pacemaker implantation, with no significant differences observed in mortality and freedom from AA at 1 and 2 years.

**The PROSPERO registration:**

CRD42024570022, https://www.crd.york.ac.uk/PROSPERO/view/CRD42024570022.

## 1. Introduction

Atrial fibrillation (AF) is the most common type of heart arrhythmia and carries significant clinical implications due to its association with increased cardiovascular mortality and thromboembolic events. It is well-established that stand-alone AF increases the risk of ischemic stroke by 2.4- to 5-fold [1, 2]. AF frequently coexists with hemodynamically significant mitral valve disease, occurring in 30–84% of such patients.

Concomitant surgical ablation remains the most effective treatment for AF, with the Cox Maze IV procedure recognized as the global gold standard.

In recent years, minimally invasive mitral valve surgery (MIMVS) has gained widespread adoption. The benefits of MIMVS are well-documented, not only in numerous studies but also through structured meta-analysis. However, when performing concomitant AF surgery, the minimally invasive approach often necessitates modifications to ablation protocols and devices. This specifically involves the use of other ablation devices specially adapted for minimally invasive surgery. In addition, ablation patterns are often incomplete due to the limited field of view, which may also affect efficacy. Consequently, a comparative evaluation of its efficacy versus full sternotomy is essential. We aimed to conduct a statistical meta-analysis comparing outcomes of combined mitral valve and AF surgery via minimally invasive access versus conventional sternotomy (CS).

## 2. Methods

### 2.1 Literature Search Strategy

A systematic literature search was conducted using the following electronic databases from their inception until September 2024: Ovid MEDLINE, EMBASE, SCOPUS, and the Cochrane Central Register of Controlled Trials. To ensure a comprehensive and selective search, the following keywords were combined: “minimally invasive”, “mitral valve surgery”, “atrial fibrillation”, “concomitant ablation”, “Cox Maze procedure”, “right minithoracotomy”, “port-access”. Only full-text articles were prioritized for inclusion. The study protocol was registered in PROSPERO (International Prospective Register of Systematic Reviews; ID: CRD42024570022). Identified studies were rigorously screened based on predefined inclusion and exclusion criteria.

### 2.2 Selection Criteria

For the statistical evaluation of perioperative and long-term outcomes, concomitant ablation during MIMVS was selected as the primary focus. The inclusion of a comparison group with CS was preferred but not mandatory. To facilitate comparative analysis, we also incorporated isolated studies where the combined mitral valve surgery and ablation procedure was performed exclusively via sternotomy.

### 2.3 Exclusion Criteria

Studies, that didn’t assess freedom from atrial arrhythmia (AA) were excluded. We also excluded:

• Procedures involving additional interventions on the aorta, aortic valve, or coronary vessels;

• Non-English language publications;

• Case reports, narrative reviews, perspective trials, conference abstracts, and studies lacking perioperative/postoperative outcome data.

Two independent reviewers evaluated all studies for inclusion. After initial screening, full-text articles were assessed for final eligibility. Any discrepancies between reviewers were resolved through consensus discussion with a third investigator.

### 2.4 Study Endpoints

The main objective was to compare procedural efficacy (freedom from AA) and safety (30-day mortality) of mitral valve surgery with concomitant surgical ablation performed via minimally invasive cardiac surgery (MICS group) versus conventional sternotomy (CS group).

The primary endpoint was 1- and 2-years freedom from any atrial arrhythmia recurrence (atrial fibrillation, atrial flutter, or atrial tachycardia) lasting more than 30 seconds, as documented by cardiac rhythm recording (12-lead electrocardiogram (ECG) or Holter monitoring). These timepoints were selected based on preliminary literature analysis of available data.

The following secondary endpoints included: assessment of 30-day mortality and the rate of postoperative pacemaker implantation. Additionally, the association between freedom from AA recurrence and the type of ablation lesion set (biatrial Maze\left-sided set) was assessed. Mean cardiopulmonary bypass (CPB) time and aortic cross-clamp (ACC) time were also evaluated as operative outcomes.

### 2.5 Statistical Analysis

**Fig. 1.**
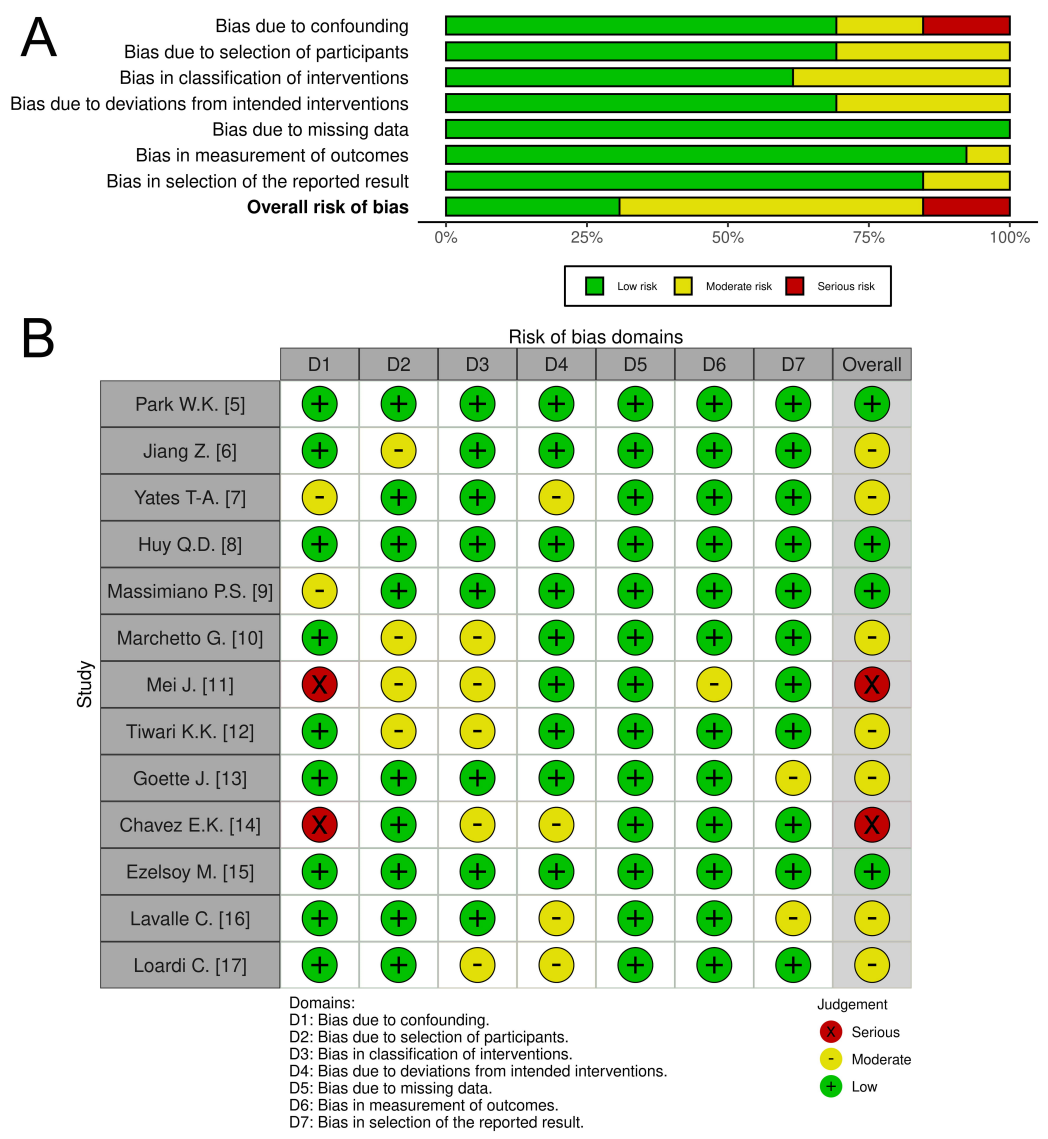
**The quality assessment of included studies**. Comments: (A) Risk of bias graph: Illustrates the distribution of bias judgments (low, moderate, serious, critical) across all assessed domains, presented as percentages. (B) Risk of bias summary: Provides a detailed breakdown of bias assessments for each individual study.

The included studies reported baseline characteristics either as mean ± standard deviation (SD) or median with interquartile range (IQR). For consistency in analysis, we converted median values to means using the established method by Wan *et al*. [3]. All outcomes were visualized using forest plots stratified by surgical approach. In order to compare continuous outcomes between the MICS and CS, we employed linear meta-regression models with random effects. For binary outcomes, logistic meta-regression models with random effects were used. The article DOI served as the sole random effect, meaning different groups from the same study shared the same effect value. The risk of bias in the included studies was assessed using the ROBINS-I tool [4]. Seven domains of bias were evaluated, and each study was subsequently classified into one of four risk categories: low, moderate, serious, or critical (Fig. 1, Ref. [5, 6, 7, 8, 9, 10, 11, 12, 13, 14, 15, 16, 17]).

To perform sensitivity analysis, we conducted a meta-analysis of comparative studies using random-effects models to account for potential heterogeneity between studies. Binary outcomes (freedom from AA, pacemaker implantation, and mortality) were analyzed using odds ratios (OR) with the Mantel-Haenszel inverse-variance method. Continuous outcomes (CPB and ACC times) were analyzed using mean differences (MD) with inverse-variance weighting. Heterogeneity was assessed using I^2^ and τ^2^ statistics. All analyses were conducted in R (version 4.3.1, R Foundation for Statistical Computing, Vienna, Austria) with the meta package, and results were visualized using forest plots. Results are reported in accordance with PRISMA guidelines.

## 3. Results

### 3.1 Study Selection

**Fig. 2.**
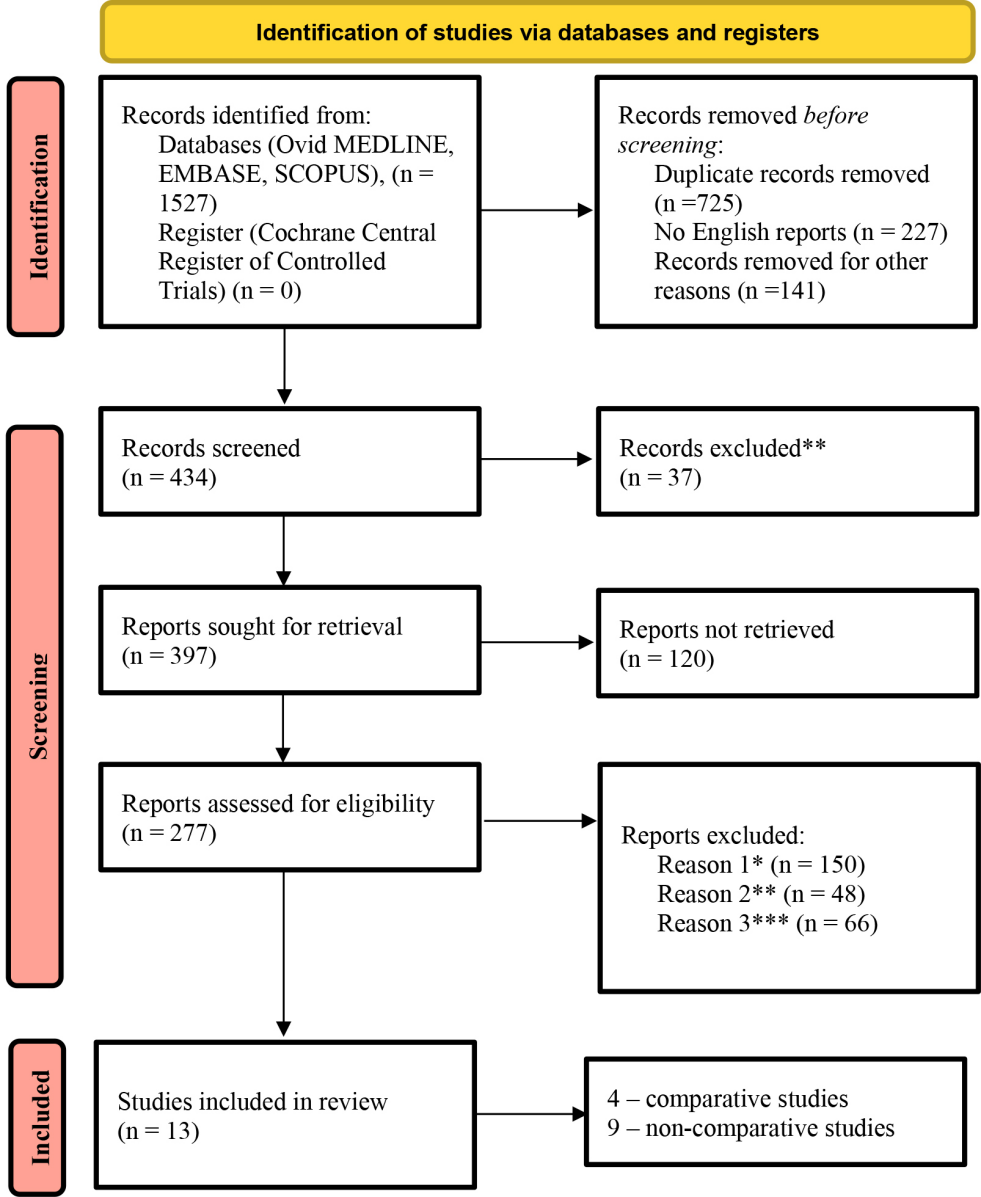
**PRISMA flow diagram of included studies**. Comments: *existence of concomitant cardiac surgeries (aortic valve surgery, aortic surgery, coronary artery bypass grafting (CABG)), **lack of data on primary and secondary endpoints, ***lack of sufficient data regarding baseline patient characteristics.

A total of 1527 potentially relevant articles were identified after searching electronic databases. Following a detailed screening, all duplicates and irrelevant studies were removed. Following rigorous application of our predefined selection and exclusion criteria, 13 studies met all eligibility requirements for inclusion in our meta-analysis. These studies collectively comprised a pooled cohort of 1216 patients (Fig. 2).

### 3.2 Study Characteristics

Detailed baseline characteristics data are presented in Table 1 (Ref. [5, 6, 7, 8, 9, 10, 11, 12, 13, 14, 15, 16, 17]). Follow-up data are presented in **Supplementary Table 1**. This review includes 4 comparative studies directly evaluating minimally invasive versus conventional sternotomy approaches. Additionally, 9 non-comparative studies examined each surgical approach separately.

**Table 1.** **Baseline characteristics**.

First author	Year	Study period	Access	Number of patients	Mean age	Male (%)	LVEF (%)	LAD (mm)	Mean duration of AF (years)	Etiology (%)			Study characteristic
										Degenerative	Rheumatic	Other	
Park W.K. [5]	2012	2006–2009	MICS	78	53.7 ± 12.2	37.2	55.8 ± 8.1	58.1 ± 9.2	5.0 ± 6.7	28.2	69.2	2.5	Port-access versus CS in patients who underwent biatrial cryoablation set.
	2012	2006–2009	CS	57	60.7 ± 10.6	43.8	57.2 ± 6.6	61.8 ± 11.7	9.3 ± 8.5	45.6	49.1	5.3	
Jiang Z. [6]	2018	2010–2015	MICS	69	60.7 ± 5.5	37.7	52.2 ± 5.1	48.6 ± 4.7	5.2 ± 2.5	36.18	63.8	0	Right minithoracotomy (RMT) versus conventional sternotomy using Cox-Maze IV ablation set with entirely bipolar radiofrequency clamp.
	2018	2010–2015	CS	83	61.7 ± 5.7	47.0	51.0 ± 4.6	49.5 ± 4.8	5.9 ± 2.6			0	
Yates T-A. [7]	2023	2004–2021	MICS	116	64.6 ± 11.8	50.0	58.4 ± 10.0	4.9 ± 1.1	4.8 ± 6.0	84	14	2.07	Propensity score matching study for concomitant mitral valve surgery and Cox-Maze procedure.
	2023	2004–2021	CS	116	65.7 ± 11.5	48.0	55.8 ± 11.3	5.4 ± 1.1	3.7 ± 5.4	61	29	8.85	
Huy Q.D. [8]	2023	2019–2022	MICS	37	53.2 ± 9.1	29.7	57.5 ± 7.9	54.8 ± 7.4	-	0	100.0	0	Port-access versus CS for long-standing persistent rheumatic AF combined with mitral valve surgery.
	2023	2019–2022	CS	44	54.7 ± 8.0	15.9	57.2 ± 10.2	52.9 ± 6.4	-	0	100.0	0	
Massimiano P.S. [9]	2013	2007–2012	MICS	34	61.3 ± 9.9	85.0	58.5 ± 9.1	-	-	-	-	-	Concomitant operation through RMT in fibrillating heart surgery.
Marchetto G. [10]	2016	2006–2014	MICS	68	65.9 ± 11.1	50.0	56.5 ± 10.4	65.3 ± 9.7	2.2	54.4	23.5	22.1	Video-assisted concomitant operation through RMT by cryoablation device.
Mei J. [11]	2016	2012–2014	MICS	59	60.9 ± 5.9	63.3	-	-	5.5 ± 2.1	-	-	-	Concomitant Maze IV ablation by bipolar radiofrequency clamp through RMT.
Tiwari K.K. [12]	2016	2012–2013	MICS	75	66.7 ± 9.8	42.7	55.3 ± 7.8	48.5 ± 7.3	2.1 ± 1.9	-	-	-	Concomitant procedure by mono- or bipolar- radiofrequency ablation.
Goette J. [13]	2016	2009–2012	MICS	60	68 ± 9	63.0	-	51 ± 9	5.3 ± 8	-	-	-	Comparative analysis of two cryoablation devices (N20 and Argon) in concomitant mitral valve surgery.
			MICS	60	67 ± 11	67.0	-	52 ± 7	4.7 ± 6	-	-	-	
Chavez E.K. [14]	2017	2013–2014	CS	103	50.8 ± 10.7	24	58.3 ± 11.7	56 ± 8.0	39.9 ± 4.7 months	0	100.0	0	Surgical treatment of AF in patients with isolated rheumatic mitral valve disease.
Ezelsoy M. [15]	2019	2001–2015	CS	68	55.6 ± 7.4	33.8	53.5 ± 6.3	53 ± 4.0	-	-	-	-	Comparative analysis of monopolar versus bipolar radiofrequency ablation in mitral valve surgery.
			CS	99	58.0 ± 6.4	44.4	54.0 ± 6.1	53 ± 5.0	-	-	-	-	
Lavalle C. [16]	2021	2008–2017	CS	100	65 ± 12	36	55.9 ± 11	52 ± 9.2	30.8 ± 1.6 months	-	-	-	Comparative analysis of left atrial appendage exclusion in patients who underwent MVS and surgical ablation.
Loardi C. [17]	2015	2005–2012	CS	122	62 ± 8.5	48.4	57 ± 9	56 ± 12	28.3 months	-	-	-	Atrial contractility after concomitant MVS and surgical ablation.

Abbreviations: AF, atrial fibrillation; LVEF, left ventricular ejection fraction; LAD, left atrium diameter; MVS, mitral valve surgery; RMT, right minithoracotomy; MICS, minimally invasive; CS, conventional sternotomy. (The studies with a direct comparison group are highlighted in color).

### 3.3 Study Endpoints

#### 3.3.1 Freedom From AA for 1 Year

**Fig. 3.**
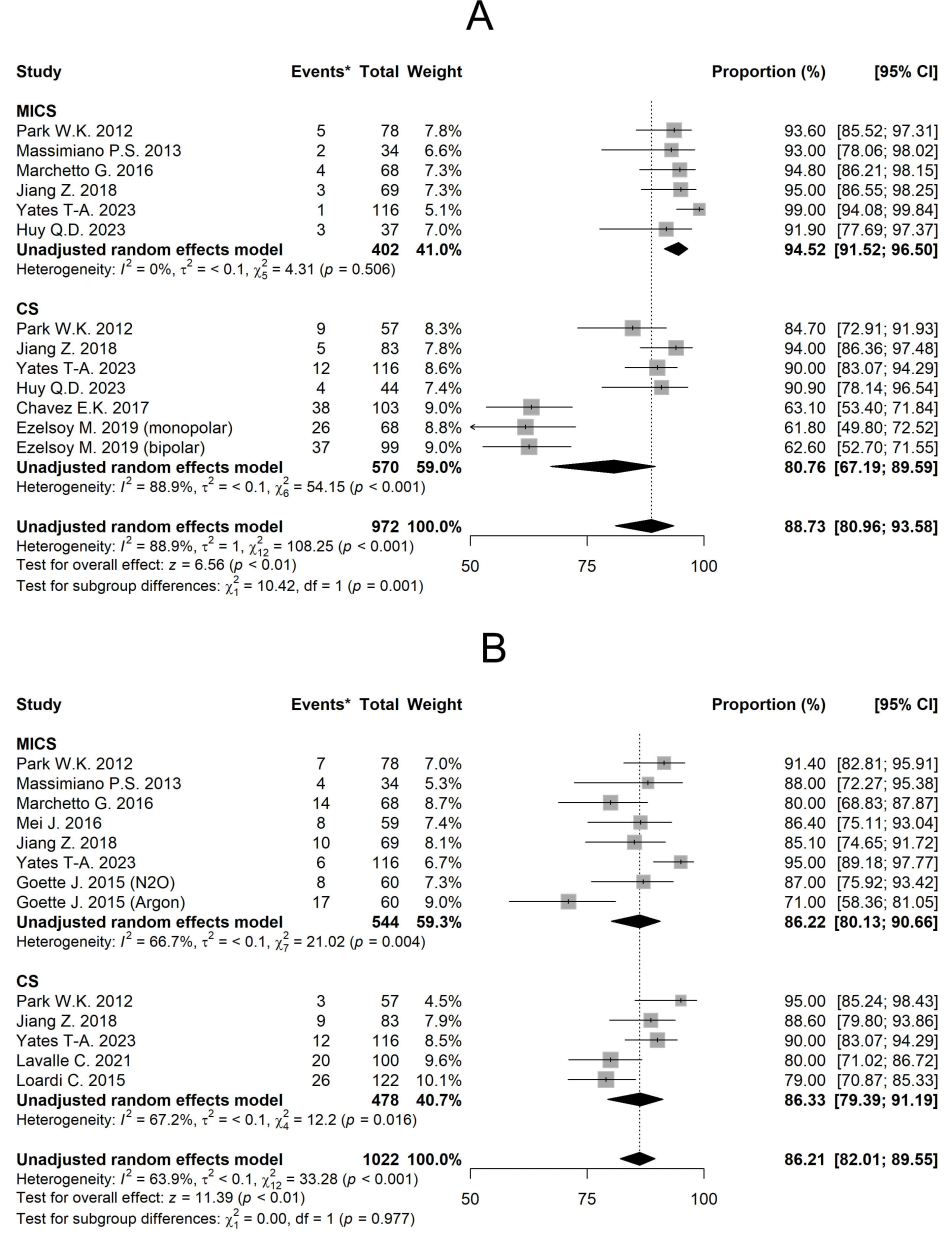
**Forest plots diagram of the Freedom from AA**. (A) Freedom from AA for 1 year. (B) Freedom from AA for 2 years. Comments: *The ‘Event’ column shows the number of patients in each study who experienced arrhythmia recurrence. MICS, minimally invasive; CS, conventional sternotomy; CI, confidence interval; AA, atrial arrhythmia.

The mean freedom from AA for 1 year of MICS and CS was 94.52% [95% CI 91.52, 96.50] and 80.76% [95% CI 67.19, 89.59] respectively (Fig. 3A). Linear meta-regression results for all outcomes are presented in Table 2, with MICS as the baseline reference. Considering the moderators, no statistically significant differences between groups were found (*p* = 0.95).

**Table 2.** **Meta-regression results for: 1- and 2-years freedom from AA, 30-day mortality, pacemaker implantation, cardiopulmonary bypass time and aortic cross-clamp time**.

Comparative and non-comparative studies				Comparative studies		
Predictors	OR	95% CI	*p*-value	OR	95% CI	*p*-value
1-year freedom from AA						
CS	1.27	0.00, 1285.42	0.951	0.32	0.13, 0.78	0.012
Maze 4	0.41	0.00, 69.48	0.734	1.00	0.89, 1.12	0.944
LAD	1.01	0.86, 1.20	0.862	0.98	0.96, 1.00	0.094
AF duration	0.67	0.05, 8.86	0.761	1.10	0.80, 1.51	0.539
2-years freedom from AA						
CS	0.82	0.44, 1.52	0.531	0.81	0.43, 1.55	0.533
Maze 4	1.02	1.01, 1.03	<0.001	0.89	0.81, 0.98	0.018
LAD	1.01	1.00, 1.02	0.183	1.01	0.99, 1.02	0.317
AF duration	1.02	0.98, 1.05	0.395	1.27	0.91, 1.78	0.153
30-day mortality						
CS	1.36	0.27, 6.88	0.709	1.02	0.20, 5.11	0.984
Pacemaker implantation						
CS	6.21	2.30, 16.79	<0.001	5.65	2.10, 15.23	0.001
Cardiopulmonary bypass time						
Predictors	Difference	95% CI	*p*-value	Difference	95% CI	*p*-value
CS	–27.46	–30.92, –23.99	<0.001	–27.31	–30.79, –23.83	<0.001
Aortic cross-clamp time						
CS	–25.72	–29.19, –22.25	<0.001	–25.8	–29.28, –22.32	<0.001

Abbreviations: AA, atrial arrhythmia; AF, atrial fibrillation; CI, confidence interval; CS, conventional sternotomy; LAD, left atrium diameter; OR, odds ratios.

#### 3.3.2 Freedom From AA for 2 Years

The mean of freedom from AA for 2 years of MICS and CS was 86.22% [95% CI 80.13, 90.66] and 86.33% [95% CI 79.39, 91.19] respectively (Fig. 3B). Meta-regression analysis again showed no significant intergroup difference (*p* = 0.531), with MICS as reference (Table 2). In addition, the use of a biatrial ablation set significantly increases the duration of AF-free survival compared to left-sided set (*p* < 0.001).

#### 3.3.3 Cardiopulmonary Bypass Time

The mean cardiopulmonary bypass time was 151.50 min (130.28–172.72) for MICS and 120.01 min (106.16–133.86) for CS (**Supplementary Fig. 1A**). Adjusted analysis revealed statistically significant differences between groups (*p* < 0.001) (Table 2).

#### 3.3.4 Aortic Cross-Clamp Time

The mean aortic cross-clamp time was 112.36 min (87.87–136.85) for MICS and 101.43 min (80.00–122.86) for CS (**Supplementary Fig. 1B**). Adjusted analysis demonstrated statistically significant intergroup differences (*p* < 0.001) (Table 2).

#### 3.3.5 Mortality

**Fig. 4.**
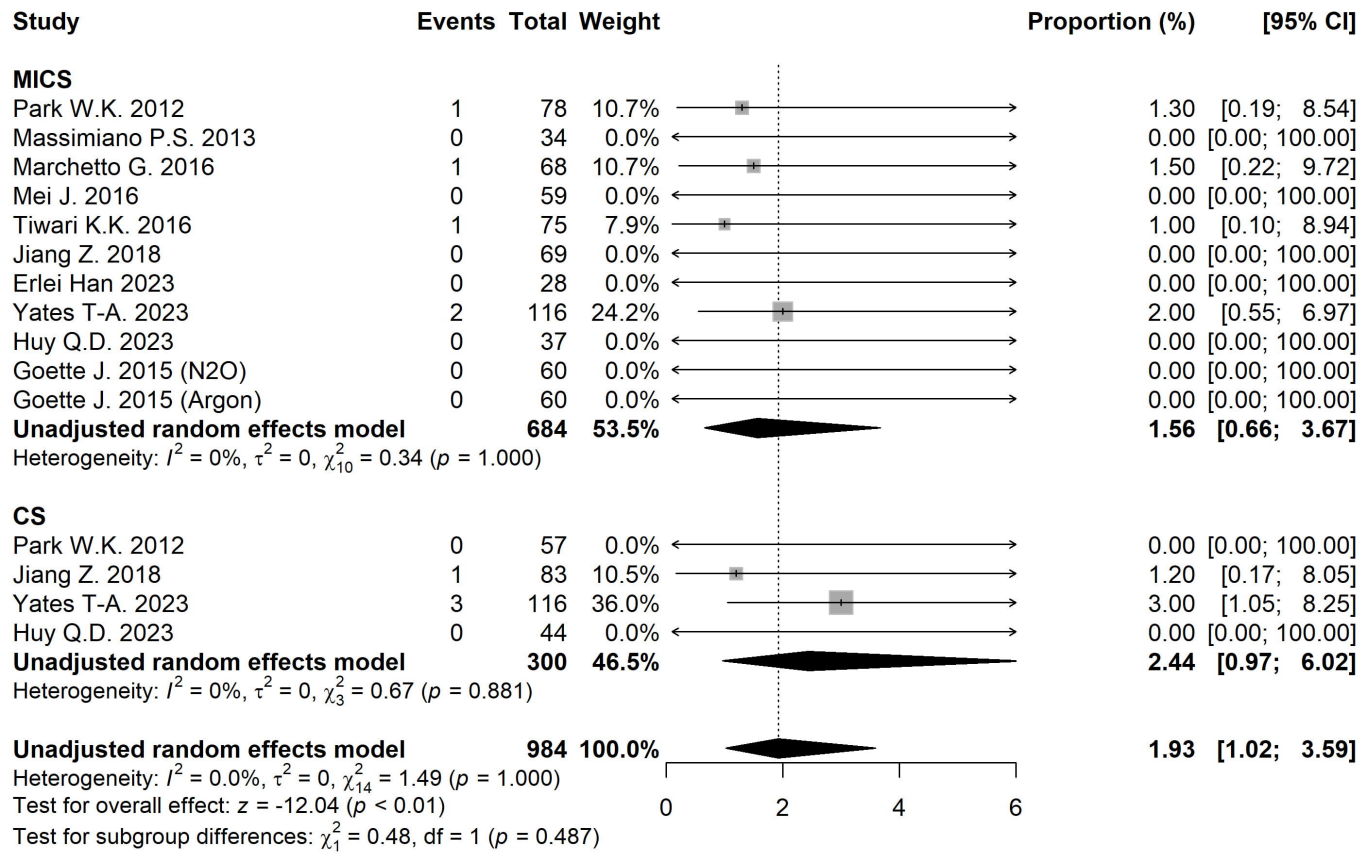
**Forest plot diagram of the 30-day Mortality**. MICS, minimally invasive; CS, conventional sternotomy; CI, confidence interval.

The 30-day mortality rate was 1.56% [95% CI 0.66, 3.67] for MICS and 2.44% [95% CI 0.97, 6.02] for CS (Fig. 4). Adjusted analysis showed no statistically significant differences between groups (*p* = 0.709) (Table 2).

#### 3.3.6 Pacemaker Implantation

**Fig. 5.**
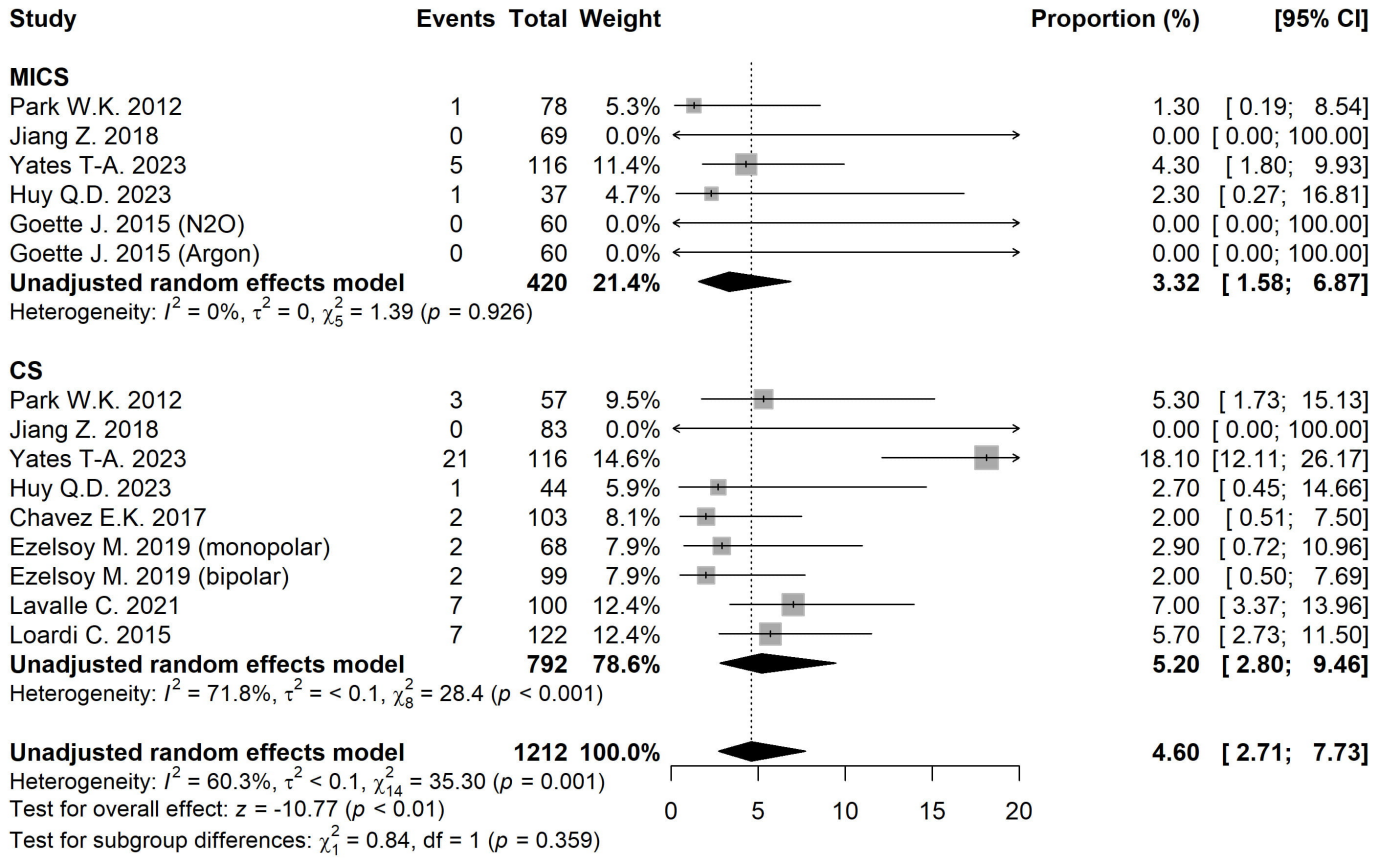
**Forest plot diagram of the Pacemaker implantation**. MICS, minimally invasive; CS, conventional sternotomy; CI, confidence interval.

The pacemaker implantation rate was 3.32% [95% CI 1.58, 6.87] for MICS and 5.20% [95% CI 2.80, 9.46] for CS (Fig. 5). After adjusting for moderators, statistically significant intergroup differences were observed (*p* < 0.001) (Table 2).

### 3.4 Sensitivity Analysis

In this type of research, addressing the heterogeneity of the included studies remains a major challenge and may hinder the ability to draw robust conclusions. To complement the primary analysis, we conducted a subanalysis limited to studies that directly compared the treatment groups [5, 6, 7, 8]. Unlike the main meta-regression analysis, this subanalysis revealed a significant difference in 1-year freedom from atrial arrhythmias, favoring the minimally invasive group (OR 0.32, 95% CI: 0.13, 0.78; *p* = 0.012). For the other outcomes, no substantial differences were observed between the main analysis and the subanalysis, as shown in Table 2.

## 4. Discussion

At present, there are several studies that have compared outcomes between minimally invasive and standard approaches for isolated mitral valve interventions. The MIMVS approach has demonstrated statistically significant reductions in postoperative pain, intra- and postoperative blood loss, intensive care unit stay, and overall hospitalization duration, while maintaining equivalent surgical efficacy [18, 19, 20, 21, 22, 23]. Regarding stand-alone surgical ablation for AF, some studies found no significant differences in freedom from atrial tachyarrhythmias between conventional sternotomy and right minithoracotomy (RMT) at 1 year [96% (97/101) vs. 92% (90/98), *p* = 0.246], 5 years [86% (42/49) vs. 93% (39/42), *p* = 0.331], and 10 years [84% (21/25) vs. 88% (7/8), *p* = 1.000] [24]. Another study group reported similar findings, with no intergroup differences in AF-free survival except at 6 months (86% (CS) vs. 75% (RMT), *p* = 0.04). Moreover, the minimally invasive group showed significantly lower rates of overall complications and 30-day mortality [25].

Based on the collective evidence from these studies, an important clinical question emerges: Should atrial fibrillation in patients with hemodynamically significant mitral valve disease be considered a contraindication for minimally invasive surgery? Our meta-analysis results demonstrate comparable arrhythmia-free survival rates between approaches at both 1-year (*p* = 0.95) and 2-year follow-ups (*p* = 0.531).

In minimally invasive procedures, cardiac access and purse-string suture placement are performed after initiating cardiopulmonary bypass, which inherently leads to differences in CPB duration (+27.46 min, *p* < 0.001). Furthermore, positioning of the ablation device requires additional time due to the restricted surgical field in minimally invasive approaches, making increased aortic cross-clamp time an expected outcome (+25.72 min, *p* < 0.001).

A particularly noteworthy finding was the significantly higher rate of permanent pacemaker (PPM) implantation in the CS group compared to the MICS group (OR 6.21, *p* < 0.001). Among all included studies, the most pronounced difference was reported by Tari-Ann Yates *et al*. [7], with PPM implantation rates of 18% (CS) versus 4% (RMT). While the authors primarily attributed this disparity to a higher incidence of sick sinus syndrome in the CS group, several other factors merit consideration. Notably, preoperative AF duration, older age, New York Heart Association (NYHA) Class III/IV, and concomitant tricuspid valve surgery have all been identified as independent predictors of postoperative PPM requirement [26, 27, 28]. Furthermore, technical differences between approaches—including variations in ablation techniques and venous cannulation methods—may contribute to the observed discrepancy in PPM implantation rates. Prospective studies controlling for patient characteristics and surgical technique variables are needed to validate these findings.

While our meta-analysis demonstrates comparable arrhythmia-free outcomes between minimally invasive and conventional approaches for combined mitral valve surgery and atrial ablation, these findings should not be interpreted as supporting universal application of minimally invasive techniques. The surgical approach must be individualized, with careful consideration of: patient comorbidities, anatomical characteristics and surgeon experience [29, 30].

## 5. Limitation

To the best of our knowledge, this is the first systematic review and meta-analysis comparing outcomes of combined mitral valve surgery and surgical AF ablation between minimally invasive and standard approaches. However, several limitations warrant consideration. The paucity of direct comparative studies necessitated inclusion of isolated cohort studies, introducing heterogeneity in patient populations, surgical protocols, and study designs. In this type of study, it is also impossible to avoid variations in the ablation devices used or differences in the ablation protocols. In such cases, meta-regression can help account for heterogeneity bias across studies.

Secondly, all included studies relied on 12-lead ECG or Holter monitoring for endpoint assessment; the lack of continuous rhythm monitoring data may affect outcome accuracy. Despite these limitations, our study provides the first comprehensive comparison of concomitant mitral valve surgery and AF ablation outcomes between surgical approaches, offering valuable insights for clinical decision-making.

## 6. Conclusion

In conclusion, comparative analysis of 13 studies demonstrates that minimally invasive and conventional approaches for patients with combined mitral valve disease and AF show comparable effectiveness in maintaining sinus rhythm at both 1-year and 2-year follow-ups. While conventional sternotomy demonstrated shorter CPB and ACC times, this approach was associated with a 6-fold increase in PPM implantation rates compared to minimally invasive techniques. Importantly, both strategies showed similar mortality outcomes. These findings suggest that the minimally invasive approach may offer particular advantages in preserving conduction system function without compromising rhythm outcomes or survival. However, the current evidence remains limited by the observational nature of included studies, underscoring the critical need for prospective randomized trials with standardized surgical and follow-up protocols to definitively establish optimal treatment strategies.

## Data Availability

All the necessary data is already presented in the study.

## Abbreviations

AA, atrial arrhythmia; ACC, aortic cross-clamp; AF, atrial fibrillation; CPB, cardiopulmonary bypass; CI, confidence interval; CS, conventional sternotomy; IQR, interquartile range; MIMVS, minimally invasive mitral valve surgery; PPM, permanent pacemaker; SD, standard deviation.

## Supplementary Material

Supplementary material associated with this article can be found, in the online version, at https://doi.org/10.31083/RCM39706.
