# Supplementary material for: Minimally Invasive or Conventional Sternotomy for Mitral Valve Surgery With Concomitant Surgical Ablation for Atrial Fibrillation: A Comparative Systematic Review
Source: Rev Cardiovasc Med. 2025 Aug 21;26(8):39706. doi: 10.31083/RCM39706 (PMC12415765; doi:10.31083/RCM39706)
Supplement: Supplementary file 1 [file 2153-8174-26-8-39706-s1.zip › Supplementary Fig. 1.docx]

***Supplementary Fig. 1:*** *Forest plot diagram of the «Artificial circulation»*

***A:*** *«Cardiopulmonary bypass time»*


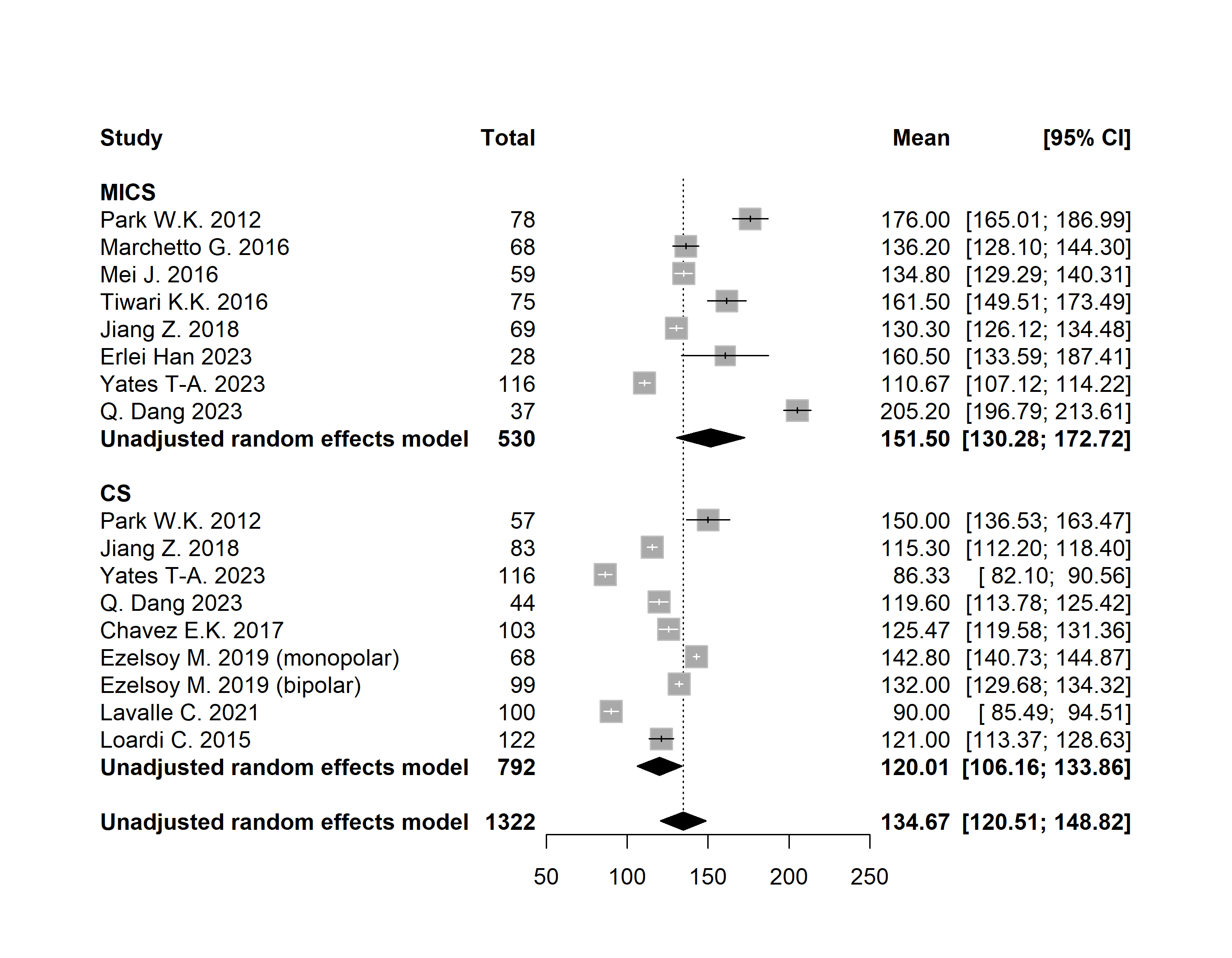

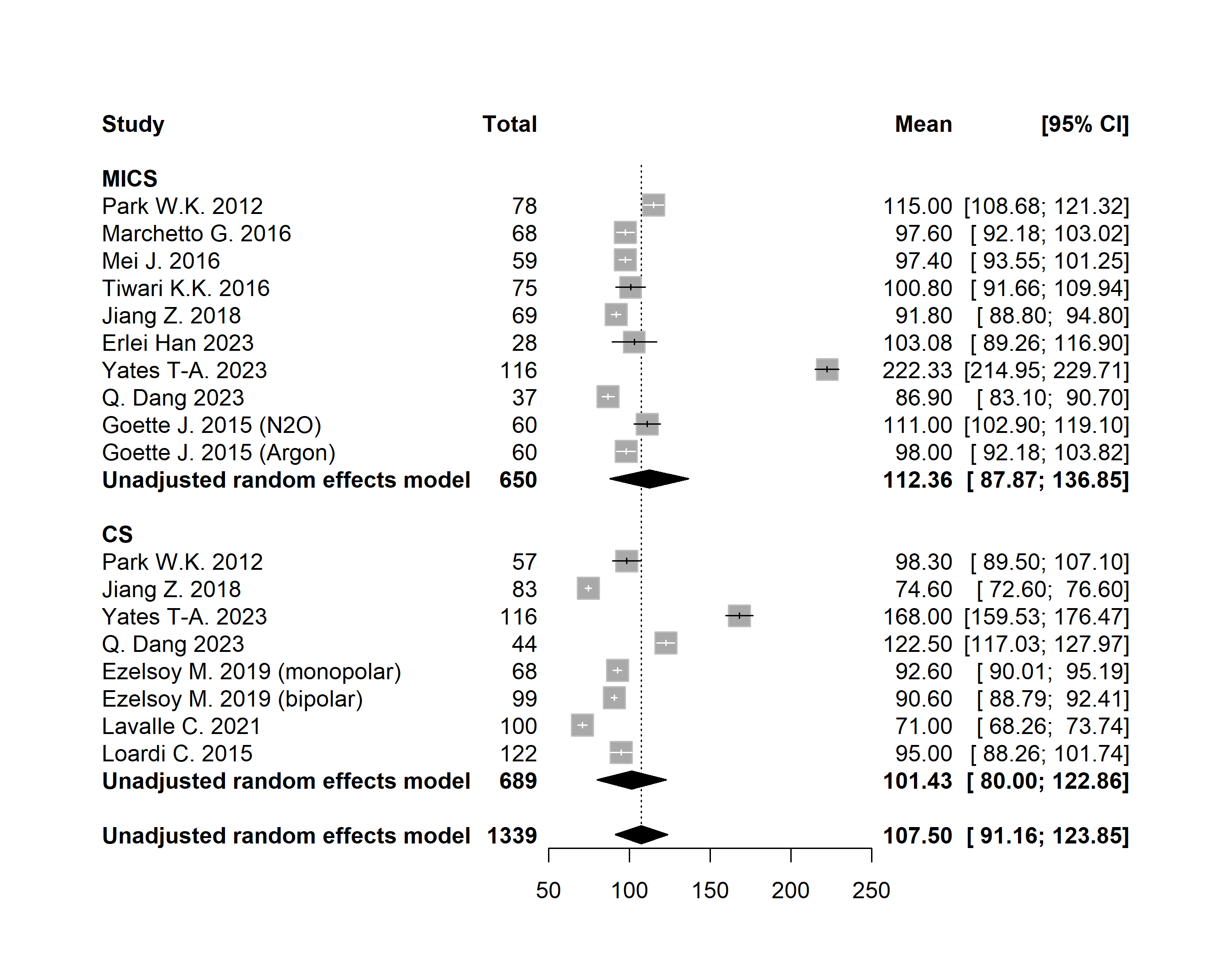


***B:*** *«Aortic cross-clamp time»*
