# Supplementary material for: Minimally Invasive or Conventional Sternotomy for Mitral Valve Surgery With Concomitant Surgical Ablation for Atrial Fibrillation: A Comparative Systematic Review
Source: Rev Cardiovasc Med. 2025 Aug 21;26(8):39706. doi: 10.31083/RCM39706 (PMC12415765; doi:10.31083/RCM39706)
Supplement: Supplementary file 1 [file 2153-8174-26-8-39706-s1.zip › Supplementary Table 1.docx]

**Supplementary Table *1*. Follow-up *data.***

| **First author** | **Access** | **Rhythm assessment** | **Freedom from**  **AA in 1 year (%)** | **Freedom from**  **AA in 2 year (%)** | **Pacemaker (%)** | **Hospital LOS (mean)** | **30-mortality (%)** |
| --- | --- | --- | --- | --- | --- | --- | --- |
| Park W.K. [4] | MICS | ECG and 24-h holter | 93.6 | 91.4 | 1.3 | - | 1 |
|  | CS |  | 84.7 | 95.0 | 5.3 | - | 0 |
| Jiang Z. [5] | MICS | ECG and 24-h holter | - | 85.1±5.8 | 0 | 9.3 ± 2.6 | 0 |
|  | CS |  | - | 88.6±3.6 | 0 | 11.7 ± 3.0 | 1 |
| Yates T-A. [6] | MICS | ECG and 24-h holter | 99.0 | 95.0 | 4.3 | 8 (6–11) | 2 |
|  | CS |  | 90.0 | 90.0 | 18.1 | 10 (7–14) | 3 |
| Q. Dang [7] | MICS | ECG and 24-h holter | 91.9 | - | 2.3 | 16.1 ± 7.6 | 0 |
|  | CS |  | 90.9 | - | 2.7 | 12.1 ± 4.4 | 0 |
| Massimiano P.S. [8] | MICS | ECG and 24-h holter | 93.0 | 88.0 | - | 5.9 | 0 |
| Marchetto G. [9] | MICS | ECG and 24-h holter | 94.8 | 80.0 | 3.3 | 10.2 ± 7.5 | 1 |
| Mei J. [10] | MICS | - | 91.5 | 86.4 | 0 | - | 0 |
| Tiwari K.K. [11] | MICS | 24-h holter | - | 63.0 | 2.7 | - | 1 |
| Goette J. [12] | MICS | ECG and 24-h holter | - | 87 | 0 | 9.8 ± 4.7 | 0 |
|  | MICS |  | - | 71 | 0 | 10.5 ± 4.8 | 0 |
| Chavez E.K. [13] | CS | ECG and 24-h holter | 63.1 | - | 2 | - | 7.8 |
| Ezelsoy M. [14] | CS | ECG and 24-h holter | 61.8 | - | 2.9 | 6.9 ± 0.7 | - |
|  | CS |  | 62.6 | - | 2.0 | 6.9 ± 0.7 | - |
| Lavalle C. [15] | CS | ECG and 24-h holter | - | 80 | 7.0 | 12 days | - |
| Loardi C. [16] | CS | 24-h holter | - | 79 | 5.7 | - | 0.8 |

**Abbreviations**: AA – atrial arrhythmia, ECG – electrocardiogram, LOS – length of stay. (The studies with a direct comparison group are highlighted in color.)
